# Mean Platelet Volume in Patients with Obstructive Sleep Apnea and Its Relationship with Simpler Heart Rate Derivatives

**DOI:** 10.1155/2014/454701

**Published:** 2014-09-10

**Authors:** Aydın Akyüz, Dursun Çayan Akkoyun, Mustafa Oran, Hasan Değirmenci, Recep Alp

**Affiliations:** ^1^Department of Cardiology, Faculty of Medicine, Namık Kemal University, 59100 Tekirdağ, Turkey; ^2^Department of Internal Medicine, Faculty of Medicine, Namık Kemal University, 59100 Tekirdağ, Turkey; ^3^Department of Neurology, Faculty of Medicine, Namık Kemal University, 59100 Tekirdağ, Turkey

## Abstract

Some studies show increased mean platelet volume (MPV) in obstructive sleep apnea (OSA). The aim of this study was to evaluate MPV in OSA patients without cardiovascular risk factors and the possible association of heart rate derivatives with MPV. A total of 82 patients (aged 30–70 years) were divided into 2 groups according to the presence of either OSA or non-OSA as the control group. The OSA group consisted of 52 patients and the control group consisted of 30 subjects. Neither group was significantly different in terms of MPV values as well as heart rate (HR) derivatives such as minimum HR, maximum HR, the difference between maximum HR and minimum HR, mean HR, and heart rate performance index (HRPI) [(HR max. − HR min.)/HR mean] (*P* > 0.05 for all variables). In multivariate analysis, platelet count and percentages of recording time spent at arterial oxygen saturation < 90% significant variables are associated with MPV (*β* ± SE: −0.004 ± 0.002, 95% CI, −0.008 to −0.001; *P* = 0.034) and (*β* ± SE: 2.93 ± 1.93, 95% CI, 0.167 to 5.69; *P* = 0.038). Consequently, our findings predominantly suggest that there is a casual and reciprocal interaction between MPV and autonomic activation.

## 1. Introduction

Recently, some studies suggest increased mean platelet volume as a platelet dysfunction in obstructive sleep apnea (OSA) syndrome [1, 2], which is associated with sympathetic stimulation due to hypoxic spells during sleep [3]. Apnea and hypopnea episodes in OSA are associated with increased parasympathetic activity during apnea and hypopnea and increased sympathetic activity at apnea and hypopnea termination. Numerous studies show that OSA patients have overt sympathetic activity due to acute central response to apnea and hypopnea [4–6]. Although decreased heart rate variability has been well documented in OSA, little consistent evidence exists concerning simpler heart rate parameters such as resting heart rate (HR), maximal HR, and minimum HR during polysomnography (PSG) in the supine position. While resting heart or mean heart rate in 24-hour ECG indirectly reflects sympathetic activation, the difference between maximal HR and minimal HR during electrocardiogram (ECG) monitoring in 24 hours reflects chronotropic ability, also known as heart rate reserve on treadmill exercise testing [7].

Although both resting heart rate and mean platelet volume (MPV) have been demonstrated as cardiovascular risk factors [8, 9], little consistent evidence exists about the autonomic nervous activity association with platelet activation [10, 11]. Increased platelet activity assessed with MPV may mediate the association between sympathetic activation and thromboembolic events. Particularly, no studies can show whether there is relationship between simpler heart rate derivatives and MPV. Accordingly, we first aimed to investigate the possible association of heart rate derivatives with MPV and, secondly, we evaluated whether there is relation between MPV and OSA in patients who have OSA without diabetes, smoking, hypertension, or metabolic syndrome.

## 2. Materials and Methods 

### 2.1. Patients

This research was an observational retrospective and cohort study. The study initially comprised 450 consecutive subjects admitted to our neurology clinics for polysomnographic examination to detect whether or not they had OSA. Patients were excluded for a range of reasons: if they had cardiac rhythm other than normal sinus rhythm; heart failure; history of coronary artery disease (CAD); history of cerebrovascular events; diabetes mellitus; metabolic syndrome; history of smoking; hypertension (>140 and or >90 mmHg); dyslipidemia; those treated with antihypertensive or antihyperlipidemic agents; overt/active hematological disorders; and those suffering from disorders such as renal, hepatobiliary, respiratory, infectious, inflammatory, or thyroid.

All the patients were asked for medical histories in terms of history of CAD, previous thromboembolic events, durations of OSA, and current medications. Diabetes was defined as the American Diabetes Association defines it [12]. HT was diagnosed as systolic blood pressure of >140 mmHg and/or diastolic blood pressure of >90 mmHg or taking antihypertensive medication. Dyslipidemia was defined as total cholesterol (TC) ≥200 mg/dL, low-density lipoprotein cholesterol (LDL-C) ≥130 mg/dL, triglycerides (TGs) ≥150 mg/dL, and high-density lipoprotein cholesterol (HDL-C) ≤40 mg/dL, as described previously [13]. None of the patients were taking antihypertensive drugs or statins. Smoking status was accepted as current smoker or history of smoking and nonsmokers were included. The study used the remaining 82 patients (60 males, 22 females) aged 46.5 ± 11 years. They were divided into two groups according to their apnea hypopnea index (AHI). Those with an AHI score of ≥5 were put in the OSA group. Those with an AHI score of <5 were recorded as the control group. Finally, the study enrolled 52 OSA patients and 30 controls. In OSA, the patients were diagnosed with OSA with varying degrees of severity (28 mild-moderate of 5–30 AHI score and 24 severe of ≥30 AHI score).

### 2.2. Laboratory

Polysomnographic (PSG) findings were obtained using a computerized system (Embla N7000; Somnologica, Broomfield, Colorado). Respiratory parameters were analyzed automatically and then reexamined visually to ensure accuracy of the data. Cessation of airflow for ≥10 seconds was defined as apnea. A reduction in thoracic excursion of ≤50% for ≥10 seconds or oxygen desaturation of ≥3% was defined as hypopnea. AHI was calculated from the number of apnea and hypopnea events per hour of sleep. Percentages of recording time spent (PRTS) at SaO_2_ < 90% were obtained for each subjects. The heart rate parameters of all the participants were obtained by screening the minimum HR, maximum HR, and mean HR in the supine position during PSG examination. Total sleep times were also obtained from the PSG data.

The difference between maximal HR and minimum HR was calculated and then heart rate performance index (HRPI) was calculated. HRPI was obtained by dividing the mean HR by the difference of maximal HR and minimum HR. [HRPI = (HR max. − HR min.)/HR mean]. Recently, we showed it as a novel index of autonomic nervous system that correlates with left ventricle ejection fraction in heart failure in the context of 24-hour Holter ECG monitoring in our previous study [14]. Logically, the difference between maximal HR and minimum HR indirectly reflects heart rate reserve used in exercise testing. Heart rate reserve is a marker of the chronotropic ability of the heart. Mean heart rate in HRPI is used as a variation of the resting HR. Both resting HR and heart rate reserve are good markers of mortality.

The body mass indexes (BMI) of all participants were calculated as weight in kilograms divided by height in meters squared and routinely recorded in the PSG data. Both the OSA and the control groups baseline characteristics such as age, gender, status of hypertension or diabetes, the circumference of waist, duration of OSA, fasting glucose, creatinine, C-reactive protein, fibrinogen levels, hemoglobin, red blood cell distribution width (RDW), white blood cells count (WBC), neutrophil count, lymphocyte count, platelet count, low-density, lipoprotein cholesterol (LDL-C), high-density lipoprotein cholesterol (HDL-C), triglyceride concentrations, and mean platelet volume (MPV) were recorded from their medical recordings in our hospital.

Fasting antecubital venous blood samples were collected in the morning after overnight fasting using a sterile 21-gage needle syringe without stasis in our biochemistry department; Vacutainer tubes (Vacutainer, Becton, Dickinson and Company, Franklin Lakes, NJ, USA) containing dry dipotassium ethylenediaminetetraacetic acid (EDTA) were used for blood sampling and the blood samples were collected in 2 mL tubes for platelet count and MPV measurement. Common blood counting parameters were measured using Roche Sysmex XT-2000i autoanalyser (Roche Diagnostics, Paris, France) and the same commercial kits within 30 minutes of venipuncture. For all measurements, both intra- and interassay coefficients of the variations were <5%. Lipid profile was determined by standard methods. The local ethics committee approved the study.

### 2.3. Statistical Analysis

Data were analyzed using the Predictive Analysis Software Statistics 18 (SPSS Inc., Chicago, Illinois). Continuous variables were tested for normal distribution using the Shapiro-Wilk normality test. Continuous data were reported as a mean and standard deviation or median. Student's *t*-test or Mann-Whitney *U* test was used to compare the continuous variables between the two groups. Categorical variables were denoted as percentages and compared with the Chi-square test. Pearson's or Spearman's correlation coefficient tests were used to determine correlations between MPV, HRPI, and other parametric variables or nonparametric variables, respectively. Univariate and multivariate linear regression analysis were performed to determine the independent correlations of MPV. The effects of different variables on MPV were calculated using a univariate analysis for each. The variables for which the unadjusted *P* value was <0.100 in univariate analysis were adjusted into full model. A two-sided *P* value < 0.05 was considered significant and confidence interval (CI) was 95%.

## 3. Results

The OSA group included 52 patients aged 48 ± 11 years with a BMI of 32 (25–47) kg/m^2^. There were 39 males (75%) and 13 females (25%) in the OSA group. The control group included 30 subjects aged 44 ± 11 with a BMI of 28.5 (20–49) kg/m^2^. There were 21 males (70%) and 9 females (30%) in the control group. In the OSA group, the percentage of severe OSA patients and the percentage of mild-moderate OSA patients were similar (53.8% versus 46.2%, *P* > 0.05). Age, gender, and BMI of the two groups were not significantly different (*P* > 0.05). The two groups were not significantly different in terms of fasting glucose, TG, HDL-C, LDL-C, creatinine, fibrinogen, or CRP levels (all values of *P* > 0.05). No significant difference in WBC, RDW, platelet count, neutrophil, lymphocyte, and neutrophil/lymphocyte ratios was noted between the groups. MPV was similar between the two groups (8.5 ± 1.1 fl versus 8.8 ± 0.8 fl; *P* < 0.001).

**Figure 1:**
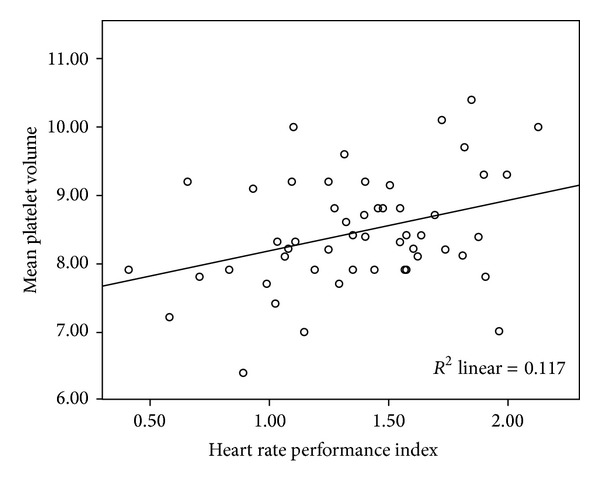
There was a positive correlation between heart rate performance index and mean platelet volume in OSA patients. (*r* = 0.343, *P* = 0.013).

**Figure 2:**
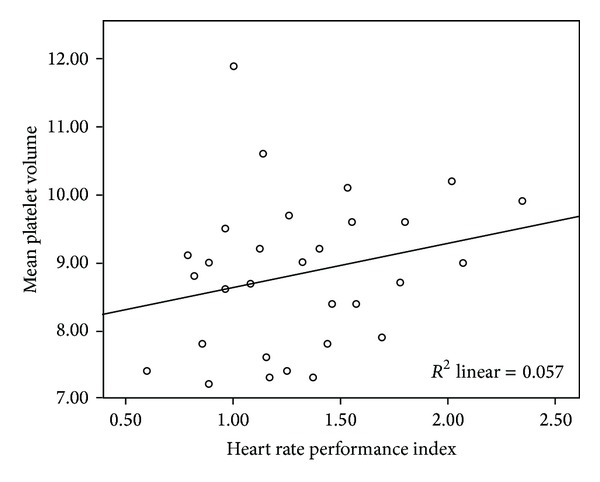
No significant correlation was seen between heart rate performance index and mean platelet volume in controls (*r* = 0.239, *P* = 0.204).

No significant difference was seen between the two groups in terms of minimum HR, maximum HR, the difference between maximum HR and minimum HR, mean HR, and HRPI or sleep time in the PSG analysis (Table 1). Surprisingly, MPV was correlated with HRPI (*r* = 0.343, *P* = 0.013) (Figure 1) and with the difference between maximum HR and minimum HR (*r* = 0.311, *P* = 0.025) and with PRTS at SaO_2_ <90% (*r* = 0.411, *P* = 0.001) in the OSA group. Else, MPV was negatively correlated with minimum HR (*r* = −0.367,*P* = 0.046) in the control group. There was no correlation between HRPI and MPV in the control group (*r* = 0.239, *P* = 0.204) (Figure 2). In addition, there was a correlation between HRPI and PRTS at SaO_2_ < 90% in the OSA group (*r* = 0.254, *P* = 0.01) (Table 2).

When we performed correlation analysis on the data of both groups in terms of MPV and heart rate derivatives, MPV was positively correlated with the difference between maximum HR and minimum HR (*r* = −0.252, *P* = 0.022) and HRPI (*r* = 0.273, *P* = 0.013) and negatively correlated with minimum HR (*r* = −0.255, *P* = 0.048) (Table 3). In addition, no correlation was seen between the fibrinogen level and AHI in the OSA group (*r* = −0.12, *P* = 0.937).

Age, BMI, fasting glucose, TG, HDL-C, LDL-C, creatinine, fibrinogen, CRP levels, WBC, RDW, platelet count, neutrophil, lymphocyte, neutrophil/lymphocyte ratio, minimum HR, maximum HR, the difference between maximum HR and minimum HR, mean HR and HRPI, sleep time, and PRTS at SaO_2_ < 90% were entered as univariate variables into regression analysis to determine the predictors of MPV. In multivariate analysis, the platelet count (*β* ± SE: −0.004 ± 0.002, 95% CI, −0.008 to −0.001; *P* = 0.034) and PRTS at SaO_2_ < 90% (*β* ± SE: 2.93 ± 1.93, 95% CI, 0.167 to 5.69; *P* = 0.038) were significant variables associated with MPV (Table 4).

## 4. Discussion

Numerous studies exist in the literature that evaluate the effects of autonomic functions of the heart in terms of heart rate variability in OSA [4–6] but little consistent evidence exists of simpler heart rate derivatives that demonstrate the autonomic effects of OSA on the heart during sleep.

We particularly focused on OSA patients with no manifest atherosclerosis or cardiovascular risk factors such as diabetes, hypertension, metabolic syndrome, dyslipidemia, and smoking. Furthermore, the mean age of our study subjects was 46.5 years old. The study revealed the following findings: patients with OSA had similar MPV values to non-OSA subjects; no significant differences were seen in terms of simpler heart rate derivatives between OSA patients and non-OSA subjects; although MPV was weakly correlated with both HRPI and the difference between maximum HR and minimum HR in the OSA group, and MPV was related to platelet count and PRTS at SaO_2_ < 90% in multivariate regression analysis. These correlations were not seen only between MPV and minimum HR in the control group, but MPV was correlated with HRPI and minimum HR and the difference between maximum HR and minimum HR in both group data analysis.

OSA is characterized by noradrenergic activation and there was a strong positive relationship between serum epinephrine level and platelet aggregation [15–18]. It has been well reported that elevated circulating epinephrine levels caused by intermittent hypoxia result in increased heart rate in OSA [15–19], and also treatment of OSAS by nasal continuous positive airway pressure therapy decreases epinephrine level and platelet aggregability and restores the physiological diurnal pattern of platelet aggregability [15, 20]. The degree of hypoxemia was documented as PRTS at SaO_2_ < 90% in the study and we found an association between PRTS at SaO_2_ < 90% and MPV. MPV was also correlated PRTS at SaO_2_ < 90% in OSA group.

The positive correlation between MPV, HRPI, and the difference between maximum HR and minimum HR potentially suggest that MPV is associated with autonomic nervous activations. While there is increased parasympathetic stimulation during apnea or hypopnea episodes, increased sympathetic activity is seen at apnea or hypopnea termination [4–6], namely, overt sympathetic and parasympathetic activity is seen in OSA. Normally, an augmentation for the difference between maximum HR and minimum HR in supine position during PSG examination in OSA patients who have no cardiac disease or diabetic autonomic neuropathy is expected, but all simpler heart rate derivativeswere similar between the groups in the study. These findings predominantly suggest that both resting HR and the difference between maximum HR and minimum HR are not good markers for the evaluation of overt sympathetic and parasympathetic activity during PSG examination in OSA patients.

Elevated mean platelet volume (MPV) has been shown as a new independent cardiovascular risk factor. MPV is an indicator of platelet activation and plays a key role in the pathophysiology of thrombotic events [21]. In previous studies, increased MPV has been demonstrated in OSA as well as in nondipper hypertension [22], acute coronary events [23], acute ischemic stroke [24], and obesity [25]. Although based upon these relationships between cardiovascular diseases and MPV, only a very limited number of studies have been conducted between MPV and OSA [1, 26], and these studies have a limited number of patients. Although our study size was small, it included OSA patients without cardiovascular risk factors such as hypertension, smoking, diabetes mellitus, metabolic syndrome, and dyslipidemia. According to our results, predictors of MPV were PRTS at SaO_2_ < 90% and platelet count. The results of the above mentioned studies are not contradicting our study findings, because they investigated either OSA patients with hypertension, diabetes mellitus, or coronary artery disease. It is well known that these diseases increase MPV value [8, 16, 27].

Fibrinogen levels were similar between OSA patients and non-OSA patients in the study and were no correlated with AHI in the OSA group. Although Wessendorf et al. [28] demonstrated that fibrinogen levels correlated with AHI and its relationship with cardioembolic events in OSA, the same fibrinogen levels between the two groups in the study could be explained by OSA patients of the study with no manifest atherosclerosis or cardiovascular risk factors such as diabetes, hypertension, metabolic syndrome, dyslipidemia, or smoking. Intermittent hypoxia causes increased hematocrit, blood viscosity, coagulation activity, and platelet activity [29], but endothelial dysfunction caused by cardiovascular risk factors is substantial for platelet activation and for increased coagulation activity [30]. Hence, our findings in OSA patients who have no substantial cardiovascular risk factors do not contradict previous studies. Our study findings potentially suggest that substantial cardiovascular risk factors such as diabetes or nondipper hypertension are needed for increased MPV values and fibrinogen levels in OSA. Intermittent hypoxia is a risk factor for endothelial dysfunction; however, concomitant cardiovascular risk factors are essential for endothelial dysfunction and atherosclerosis.

No detailed study of MPV levels have been seen in patients without substantial cardiovascular risk factors until the present study, which showed normal levels in the OSA group, possibly as a result of the absence of concomitant cardiovascular risk factors. Future studies are needed that use OSA patients without substantial cardiovascular risk factors so that their results can determine if intermittent hypoxia may contribute to increased MPV values.

Our study has some potential drawbacks. First, we had only a small number of patients with OSA. However, we identified these patients from 450 subjects who had been examined by PSG. We particularly recruited OSA patients who had no substantial cardiovascular risk factors. If we had enrolled patients with OSA who had hypertension, diabetes, and CAD, the relationship between OSA and MPV values would not have been well documented. Second, it was a retrospective study. Third, we used only MPV as a platelet function marker; if the study had used multiple markers of platelet function, such as glycoprotein IIb/IIIa, thromboglobulin, or platelet factor 4, these markers could have validated findings further. We could not use them due to specialized technique and equipment requirements.

## 5. Conclusions

In conclusion, OSA patients who have no substantial cardiovascular risk factors have not increased MPV and fibrinogen levels. In addition, OSA patients' simpler heart rate derivatives obtained during PGS are not useful for the evaluation overt sympathetic and parasympathetic activity. However, the presence of a positively correlation between MPV and HRPI in the OSA group predominantly suggests that there is a casual relation between MPV and autonomic activation. Hence, further prospective randomized studies are warranted to confirm a causal relation.

## Figures and Tables

**Table 1:** Anthropometric, clinical features, and biochemical variables of the two groups.

Variables	OSA (*N* = 52)	Controls (*N* = 30)	*P* value
Age, years	48 ± 11	44 ± 11	0.101^a^
Male, *n* (%)	39 (75)	21 (70)	0.623^c^
Body mass index, kg/m^2^	32 (25–47)	28.5 (20–49)	0.07^b^
Fasting glucose, mg/dL	91 (75–99)	89 (74–99)	0.562^b^
Triglycerides, mg/dL	126 (45–147)	124 (57–149)	0.308^b^
HDL cholesterol, mg/dL	41 (27–82)	45 (28–76)	0.175^b^
LDL cholesterol, mg/dL	119 ± 28	116 ± 29	0.818^a^
Creatinine, mg/dL	0.85 (0.5–1.4)	0.81 (0.5–1.3)	0.667^b^
C-reactive protein, mg/dL	4.3 (0.3–6.1)	3.6 (0.1–5.6)	0.166^b^
Fibrinogen, mg/dL	326 ± 71	311 ± 62	0.424^a^
Hemoglobin, mg/dL	14.5 (11–16)	14.4 (11–16)	0.606^b^
RDW	13.2 (9–19)	13.6 (12–19)	0.307^b^
White blood cells, /mm^3^	6.8 ± 1.6	7.2 ± 1.9	0.329^a^
Neutrophil/mm^3^	3.9 (2.4–5.9)	3.9 (1.3–6.6)	0.270^b^
Lymphocyte/mm^3^	2.7 (1.9–6.3)	2.5 (2.1–4.7)	0.577^b^
Neutrophil/lymphocyte	1.5 (0.5–3.4)	1.5 (0.5–4.4)	0.551^b^
Platelet count (×10^9^/L)	259 ± 62	235 ± 44.7	0.07^a^
Mean platelet volume (fL)	8.5 ± 1.1	8.8 ± 0.8	0.09^a^
Apnea hypopnea index score	29 (8–75)	1 (0–4)	<0.001^c^
SaO_2_ < 90%, PRTS	27 ± 12	1.2 ± 0.8	<0.001^a^
Mild-Moderate OSA, *n* (%)	28 (53.8)	0	<0.001^c^
Severe OSA, *n* (%)	24 (46.2)	0	<0.001^b^
Duration of OSA, years	4 ± 1	0	<0.001^a^
Minimum HR-PSG	31 (25–60)	31 (25–63)	0.465^b^
Maximum HR-PSG	131 (68–150)	124 (96–148)	0.832^b^
Maximum HR-minimum HR PSG	91 ± 23	89 ± 22	0.645^a^
Mean HR-PSG	67 ± 8	69 ± 9	0.312^a^
HRPI-PSG	1.4 ± 0.4	1.3 ± 0.4	0.494^a^
Sleep time, minutes	392 ± 36	385 ± 44	0.644^a^

Abbreviations: HDL: high-density lipoprotein; LDL: low-density lipoprotein; RDW: red blood cell distribution width; HR: heart rate (beats/minute), PRTS: percentage of recording time spent; SaO_2_: arterial oxygen saturation; PSG: polysomnography. ^a^Student's *t*-test. ^b^Mann-Whitney *U* test. ^c^Chi-square test.

**Table 2:** Correlations between HRPI, MPV, and other variables in the OSA and in the control group.

Variables	OSA group				Controls			
	HRPI		MPV		HRPI		MPV	
	*r*	*P*	*r*	*P*	*r*	*P*	*r*	*P*
Age	0.006	0.964^a^	0.001	0.994^a^	0.101	0.595^a^	−0.060	0.752^a^
BMI	0.051	0.718^b^	−0.160	0.258^b^	0.263	0.160^b^	−0.095	0.619^b^
CRP	0.112	0.449^b^	0.024	0.871^b^	0.139	0.482^b^	−0.281	0.147^b^
Fibrinogen	0.018	0.902^b^	0.090	0.546^a^	0.213	0.341^a^	−0.278	0.210^a^
Hemoglobin	0.061	0.669^b^	0.054	0.704^b^	−0.263	0.161^b^	−0.264	0.235^b^
RDW	−0.207	0.142^b^	−0.071	0.615^b^	−0.073	0.700^a^	−0.057	0.766^b^
WBC	−0.058	0.684^b^	0.144	0.310^a^	−0.296	0.112^b^	−0.093	0.626^a^
Neutrophil	−0.160	0.256^b^	0.069	0.625^b^	−0.300	0.108^b^	−0.090	0.638^b^
Lymphocyte	0.083	0.561^b^	−0.053	0.709^b^	−0.239	0.203^b^	0.062	0.582^b^
*N*/*L* ratio	−0192	0.173^b^	0.080	0.573^b^	−0.005	0.980^b^	−0.049	0.798^b^
Platelet	−0.174	0.218^b^	−0.396	0.004^b^	0.133	0.485^b^	−0.159	0.403^a^
AHI	0.199	0.157^b^	0.064	0.654^b^	0.173	0.361^b^	0.135	0.478^b^
SaO_2_ < 90%, PRTS	0.254	0.01^a^	0.411	0.001^a^	0.158	0.117^a^	0.175	0.051^a^
Min. HR	−0.529	<0.001^b^	−0.147	0.297^b^	−0.766	<0.001^b^	−0.367	0.046^b^
Max. HR	0.774	<0.001^b^	0.254	0.069^b^	0.918	<0.001^b^	0.122	0.521^b^
Min. HR–Max. HR	0.908	<0.001^a^	0.311	0.025^a^	0.896	<0.001^a^	0.215	0.253^a^
Mean HR	−0.388	0.005^a^	−0.151	0.285^a^	−0.552	0.002^a^	−0.089	0.638^a^

Abbreviations: AHI: apnea hypopnea index, *N*/*L*: neutrophil/lymphocyte, RDW: red blood cell distribution width, HR: heart rate, PRTS: percentage of recording time spent; SaO_2_: arterial oxygen saturation. ^a^Pearson correlation test. ^b^Spearman correlation test.

**Table 3:** Correlations between MPV and heart rate derivatives during polysomnography on the data of both groups.

Variables	MPV	
	*r*	*P*
Min. HR	−0.255	0.048^b^
Max. HR	0.210	0.058^b^
Min. HR–Max. HR	−0.252	0.022^a^
Mean HR	−0.098	0.380^a^
HRPI	0.273	0.013^a^

HR: heart rate. ^a^Pearson correlation test. ^b^Spearman correlation test.

**Table 4:** Independent predictors of mean platelet volume in univariate and multivariate linear regression analysis in OSA groups.

Variables	Unstandardized *β* ± SD	*t*	*P* value	95% confidence interval
Univariate				
Age	−0.003 ± 0.009	−0.317	0.752	From −0.020 to 0.015
Body mass index	−0.012 ± 0.018	−0.650	0.518	From −0.047 to 0.024
Fasting glucose	−0.021 ± 0.017	−1.219	0.227	From −0.055 to 0.013
Hemoglobin	−0.46 ± 0.078	−0.065	0.559	From −0.200 to 0.019
RDW	−0.057 ± 0.070	0.808	0.421	From −.083 to 0.196
Fibrinogen	0.001 ± 0.002	−0.090	0.546	From −0.002 to 0.005
Platelet count	−0.004 ± 0.002	−2.280	0.025	From −0.008 to 0.000
Neutrophil	0.002 ± 0.005	3.328	−0.482	From −0.012 to 0.007
Lymphocyte	0.006 ± 0.017	0.373	0.710	From −0.028 to 0.041
Neutrophil/lymphocyte	−0.181 ± 0.128	−1.415	0.161	From −0.436 to 0.074
C reactive protein	−0.004 ± 0.001	−0.400	0.690	From −0.026 to 0.017
Tryglyceride	0.000 ± 0.001	0.595	0.554	From −0.004 to 0.002
LDL-cholesterol	−0.002 ± 0.003	−0.740	0.461	From −0.008 to 0.004
HDL-cholesterol	−0.008 ± 0.01	−0.807	0.423	From −0.027 to 0.012
Min. HR-PSG	−0.014 ± 0.012	−1.157	0.251	From −0.038 to 0.010
Max. HR-PSG	0.011 ± 0.06	0.265	0.058	From 000 to 0.022
Min. HR–Max. HR-PSG	0.006 ± 0.006	1.097	0.276	From −0.05 to 0.018
Mean HR	−0.011 ± 0.013	−0.882	0.380	From −0.36 to 0.014
HRPI-PSG	0.657 ± 0.259	0.273	0.013	From 0.143 to 1.172
Apnea hypopnea index	−0.005 ± 0.005	−0.968	0.336	From −0.014 to 0.005
Duration of OSA	−0.021 ± 0.017	−1.219	0.227	From −0.055 to 0.013
SaO_2_ < 90%, PRTS	3.25 ± 1.29	2.51	0.014	From 0.681 to 5.81

Multivariate				
Platelet count	−0.004 ± 0.002	−2.190	0.034	From −0.008 to −0.001
SaO_2_ < 90%, PRTS	2.93 ± 1.39	2.11	0.038	From 0.167 to 5.69
Max. HR	0.003 ± 0.013	0.203	0.840	From −0.023 to 0.028
Min. HR–Max. HR-PSG	−0.001 ± 0.016	−0.090	0.929	From −0.035 to 0.032
HRPI-PSG	0.591 ± 0.252	0.234	0.396	From −0.797 to 1.978

Abbreviations: Min: minimum; Max: maximum; HRPI-PSG; heart rate performance index-polysomnography; OSA: obstructive sleep apnea; PRTS: percentage of recording time spent; SaO_2_: arterial oxygen saturation; RDW: red blood cell distribution width.
